# Lateral Information Processing by Spiking Neurons: A Theoretical Model of the Neural Correlate of Consciousness

**DOI:** 10.1155/2011/247879

**Published:** 2011-10-23

**Authors:** Marc Ebner, Stuart Hameroff

**Affiliations:** ^1^Wilhelm-Schickard-Institut für Informatik, Eberhard-Karls-Universität Tübingen, Abt. Cognitive Systems, Sand 1, 72076 Tübingen, Germany; ^2^Departments of Anesthesiology, Psychology and Center for Consciousness Studies, The University of Arizona, Tucson, AZ 85724, USA

## Abstract

Cognitive brain functions, for example, sensory perception, motor control and learning, are understood as computation by axonal-dendritic chemical synapses in networks of integrate-and-fire neurons. Cognitive brain functions may occur either consciously or nonconsciously (on “autopilot”). Conscious cognition is marked by gamma synchrony EEG, mediated largely by dendritic-dendritic gap junctions, sideways connections in input/integration layers. Gap-junction-connected neurons define a sub-network within a larger neural network. A theoretical model (the “conscious pilot”) suggests that as gap junctions open and close, a gamma-synchronized subnetwork, or zone moves through the brain as an executive agent, converting nonconscious “auto-pilot” cognition to consciousness, and enhancing computation by coherent processing and collective integration. In this study we implemented sideways “gap junctions” in a single-layer artificial neural network to perform figure/ground separation. The set of neurons connected through gap junctions form a reconfigurable resistive grid or sub-network zone. In the model, outgoing spikes are temporally integrated and spatially averaged using the fixed resistive grid set up by neurons of similar function which are connected through gap-junctions. This spatial average, essentially a feedback signal from the neuron's output, determines whether particular gap junctions between neurons will open or close. Neurons connected through open gap junctions synchronize their output spikes. We have tested our gap-junction-defined sub-network in a one-layer neural network on artificial retinal inputs using real-world images. Our system is able to perform figure/ground separation where the laterally connected sub-network of neurons represents a perceived object. Even though we only show results for visual stimuli, our approach should generalize to other modalities. The system demonstrates a moving sub-network zone of synchrony, within which the contents of perception are represented and contained. This mobile zone can be viewed as a model of the neural correlate of consciousness in the brain.

## 1. Introduction: Cognition and Consciousness

Cognitive brain functions including sensory perception and control of behavior are ascribed to computation in networks of neurons (“neurocomputation”). In each biological neuron, dendrites (and the cell body/soma) receive and integrate synaptic inputs to a threshold for axonal firing as output—“integrate-and-fire.” Even though the behavior of an actual biological neuron is quite complex, in replicating complex behaviors, neurons are frequently modeled as simple integrate-and-fire neurons. Neuronal firings and their chemical synaptic transmissions are presumed to act like “bit states” in silicon computers. Information flows directionally through landscapes of integrate-and-fire neurons in feed-forward and feedback networks, accounting for various forms of brain cognition [1].

What cannot be easily accounted for is consciousness. Subjective phenomenal experience—conscious awareness–does not naturally ensue from information processing [2]. Without consciousness, nonconscious cognitive processing and behaviors are performed habitually, for example, on “autopilot” [3] or in “zombie mode” [4]. Without addressing consciousness *per se*, neuroscientists aim to identify the “neural correlate of consciousness” (NCC), brain systems active concomitantly with conscious experience [1].

Cognition and consciousness may, or may not, coincide. Complex behaviors like walking or driving are at times nonconscious autopilot functions and at other times accompanied by conscious perception and control. For example, we may drive to work on nonconscious autopilot while daydreaming—our conscious minds roaming elsewhere. But if a horn sounds or a light flashes, our conscious mind returns to conscious perception and control. Studies of stimulus-independent thought (“mind wandering”) show activity literally moving around the brain as the content of consciousness changes [5].

Measurable brain activity correlating most closely with consciousness (i.e., the NCC) is synchronized electrical activity in a particular frequency band (30 to 90 Hz) of the electroencephalogram (EEG) called gamma synchrony [6, 7]. EEG signals including gamma synchrony are produced by membrane potentials reflecting integration in dendrites and cell bodies, that is, not from axonal firings. Gamma synchrony can occur locally within a brain region, between neighboring regions, or globally distributed among spatially separated brain regions.

The mechanism of long-range gamma synchrony remains unclear [8]. Melloni et al. [9] assume long-range synchronization of neural assemblies to be the key event mediating access to consciousness. Different mechanisms which could induce synchronous oscillations are reviewed by Ritz and Sejnowski [10]. Local gamma synchrony requires something other than directional axonal-dendritic or axonal-cell body neurocomputation mediated by chemical synapses and axonal firings. Local gamma synchrony depends on dendrites of neighboring neurons fused and synchronized by electrical synapses or gap junctions [11–14]. In the context of neural networks, gap junction electrical synapses form lateral or sideways connections mediating synchrony (“sideways synchrony”) in input/integration layers.

As gap junctions open and close, neuronal groups linked laterally by gap junctions—subnetworks—evolve, and can move as spatiotemporal envelopes, or zones of “sideways synchrony” through the brain's neuronal networks (as feed-forward and feedback neurocomputation continue). Such moving zones of sideways synchrony have been proposed as a mobile agent/NCC (the “conscious pilot”) conveying conscious experience and choice to otherwise nonconscious autopilot cognition [15]. Human electrophysiological studies show zones of synchrony moving through the brain with changing content of consciousness [16].

## 2. Neural Network Modeling

Artificial neural networks are used to address various technical problems, replicating human or animal behavior or for modeling brain functions. In so doing, the essential ingredients of biological neuronal function are sought, omitting aspects considered inessential. A simple model capturing all the necessary ingredients has the advantage that it can be simulated faster compared to a more elaborate model. In this paper we follow the approach of Gerstner et al. [17] who focus on the spiking behavior. The molecular interaction, that is, interactions at the level of neurotransmitters and ion channels, is not considered. However we do consider connections normally omitted as inessential: sideways or lateral interneuronal connections due to dendritic-dendritic gap junctions. Using large-scale modeling [18] this may eventually lead to a better understanding of how the brain functions. We start with integrate-and-fire neurons as basic components of artificial neural networks.

**Figure 1:**
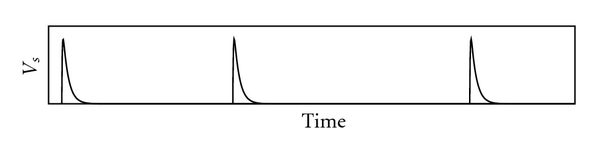
Three spikes traveling along axon.

**Figure 2:**
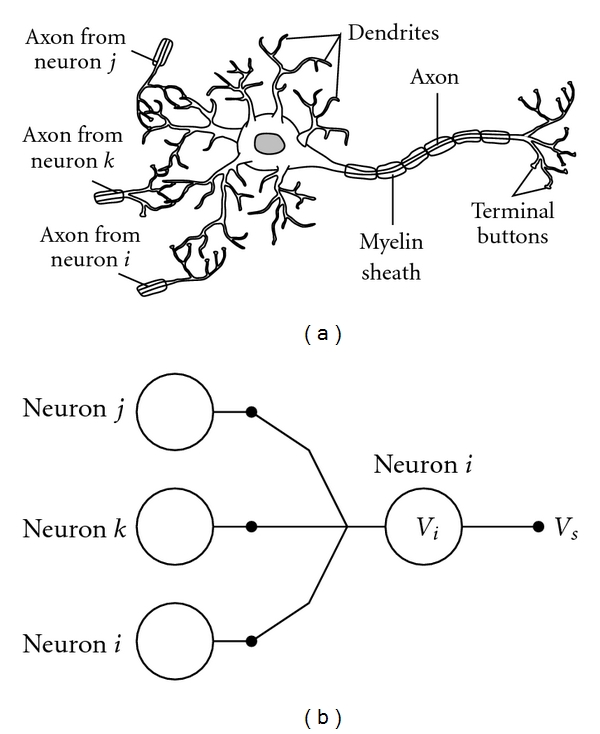
(a) Biological neuron. (b) Abstract neuron with three neuronal inputs. An abstract neuron is described by several parameters and state variables, for example, the activation or the connection weights. If the activation rises above a threshold, then the neuron sends a voltage spike along the axon which is then integrated by other neurons through its dendrites.

One of the simplest models of how a biological neuron operates is the integrate-and-fire model [19, 20]. In each biological neuron, dendrites (and the cell body/soma) receive and integrate synaptic inputs from axons of other neurons. Inputs to dendrites and cell body are integrated over time as a membrane activation potential. When the activation potential reaches a critical threshold on the proximal axon, the neuron “fires” and sends a traveling wave or spike (Figure 1) along the length of the axon to the next synapse and, hence, the next neuron. The spike is integrated, along with others from other neurons, by the next neuron. This model is shown in Figure 2.

In integrate and fire models the change of the membrane potential *V*_*i*_ of a neuron *i* which is connected to *N* other neurons is described as (modified from [21])

(1) $$C(dV_{i}/dt)=g_{i}\left(E_{i}-V_{i}\right)+I_{\text{tonic}}+I_{i}+\overset{N}{\underset{j=1}{\sum }}w_{ij}K_{j},$$

where *C* is the capacitance of the neuron. The cell tends naturally towards its resting potential *E*_*i*_. If *V*_*i*_ is higher than *E*_*i*_ then the term *g*_*i*_(*E*_*i*_ − *V*_*i*_) ensures that the membrane potential *V*_*i*_ slowly decays towards *E*_*i*_. The variable *g*_*i*_ specifies leakage conductivity, that is, the speed with which this decay occurs. The factor *I*_*i*_ takes into account that the neuron *i* may receive a constant current from an arbitrary external source. Finally, the last term ∑_*j*=1_^*N*^ *w*_*ij*_ *K*_*j*_ models the incoming current due to the excitatory potential of the incoming spike *K*_*j*_ on afferent *j*. Here, *w*_*ij*_ models the strength of the connection between neuron *i* and neuron *j*. The potential *V*_*i*_ of neuron *i* rises (*C*(*dV*_*i*_/*dt*) > 0) if *g*_*i*_(*V*_*i*_ − *E*_*i*_) > *I*_tonic_ + *I*_*i*_ + ∑_*j*=1_^*N*^ *w*_*ij*_ *K*_*j*_. Once a threshold voltage of *V*_threshold_ is exceeded, a spike is generated by the neuron *i*. The spiking voltage *V*_*s*_ is assumed to rise exponentially and also to decay exponentially.

Even though (1) is a currently accepted model of how the membrane potential of neuron *i* changes over time, it is not a particularly useful description when we want to find out which function is actually performed by neuron *i*.

## 3. A Sideways-Connected Model of Spiking Neurons

We will now gradually simplify the equation of the membrane potential in an effort to derive the function which is computed by neuron *i* and also extend this equation. First, we note that the tonic current *I*_tonic_ can be subsumed into *I*_*i*_. Hence we only need to consider cases with *I*_tonic_ = 0. The external current can be treated as another input through the afferent *j* = *N* + 1 with *w*_*ij*_ = 1. The capacitance *C* can also be removed from the equation (it results in the time constant *τ* = *C*/*g*_*i*_) by subsuming it into the constants *g*_*i*_ and the weights *w*_*ij*_. Therefore, our simplified equation describing the membrane potential *V*_*i*_ is given as

(2) $$\frac{dV_{i}}{dt}=g_{i}\left(E_{i}-V_{i}\right)+I_{i}$$

with *I*_*i*_ = ∑_*j*=1_^*N*^ *w*_*ij*_ *K*_*j*_. Using *V*_*i*_(*t* = 0) = *E*_*i*_, we obtain

(3) $$V_{i}\left(t\right)=\left(E_{i}+\frac{I_{i}}{g_{i}}\right)\left(1-\exp\left(-g_{i}t\right)\right),$$

as a solution to this equation. The membrane potential rises exponentially and reaches *E*_*i*_ + (*I*_*i*_/*g*_*i*_) for *t* → *∞* if the time between spikes is smaller than the time until the neuron has reached its resting potential. For small *t*, when *V*_*i*_ ≈ *E*_*i*_, the membrane potential rises linearly according to *V*_*i*_(*t*) = *E*_*i*_ + *I*_*i*_ *t*.

With respect to the operation of the neuron we will now consider the resting potential to be zero, that is, *E*_*i*_ = 0. Thus, we obtain

(4) $$\frac{dV_{i}}{dt}=-g_{i}V_{i}+I_{i},$$

where *g*_*i*_ defines the velocity with which the membrane voltage of the neuron returns to the resting voltage zero and *I*_*i*_ is an external input through the afferent. Let us write the above as an update equation using a time step of *dt* = 1. Let *V*_*i*_^*n*^ be the new membrane potential at the next time step which can be computed from the potential at the previous time step *V*_*i*_^*o*^. Then we obtain

(5) $$\begin{array}{cc} V_{i}^{n} & =V_{i}^{o}-g_{i}V_{i}^{o}+I_{i},\\ V_{i}^{n} & =\left(1-g_{i}\right)V_{i}^{o}+g_{i}\left(\frac{I_{i}}{g_{i}}\right),\\ V_{i}^{n} & =\left(1-g_{i}\right)V_{i}^{o}+g_{i}I_{i}^{\prime }, \end{array}$$

with *I*′ = (*I*_*i*_/*g*_*i*_). This is simply a temporal averaging operation. Suppose that *g*_*i*_ = 0.001, then this equation would simply describe that we maintain a running average of 999 previous parts *V*_*i*_^*o*^ and one part of the current input *I*_*i*_′. In other words, the main operation of the neuron is to compute a temporal average of the input *I*_*i*_′.

So far we have considered only inputs and outputs for a single integrate-and-fire neuron in a feed-forward network connected by chemical synapses. However neurons also have electrical synaptic connections mediated by structures called gap junctions [11–14] which may mediate gamma synchrony supporting conscious sensations [22–24].

Gap junctions are pores on membranes of adjacent cells composed of connexin proteins which electrically synchronize and physically fuse the two cells, forming continuous membranes and cell interiors. In the brain, gap junctions occur primarily between dendrites of neighboring neurons and mediate gamma synchrony, the best measurable correlate of consciousness. Gap junctions enable integration in dendrites of multiple neurons simultaneously, effecting collective integration. In the context of artificial neural networks, gap junctions are lateral or sideways connections in input/integration layers.

We will model gap junctions as resistive coupling between neurons [25, 26]. Two different functions are assumed to be associated with each gap junction. If a gap junction between two neurons exists, then these neurons are resistively coupled. This coupling exists unconditionally. However, we also assume a conditional coupling through gap junctions in which a particular gap junction can be in one of two modes. The gap junction can be open (electrically coupled to the neighboring neuron) or closed (electrically uncoupled from neighboring neuron) [27].

We assume that a neuron has an internal activation potential and an external membrane potential. Instead of distinguishing between internal activation potential and external membrane potential, we could also work with a compartmental model, where the two potentials are mapped to different compartments of the neuron. However, separating between internal and external makes it easier to visualize how the neuron operates. The internal activation potential (which can be measured on the inner membrane) is described by (1) or its simplified form (2). Whenever this activation potential rises above a certain threshold, the neuron fires. A spike is generated, and this spike travels down the axon of the neuron. The external membrane potential (which can be measured on the outer membrane) is influenced by the outgoing spikes and through the resistive coupling to other neurons. If a gap junction exists between two neurons, then a resistor is assumed to couple the outer membrane potential of these two neurons. The resistor connecting the outer membranes of two neurons is assumed to connect the two neurons irrespective of whether the gap junction is open or closed. Such neurons form a fixed resistive grid. This resistive grid receives as input the temporal integration of the outgoing spikes. Another resistive grid is assumed to be formed through open gap junctions. This is basically a reconfigurable resistive grid where resistors can be inserted or removed from the resistive grid by opening or closing gap junctions. The reconfigurable resistors are assumed to connect the internal activation potential to neighboring neurons allowing these neurons to fire in synchrony when gap junctions are open.

Note that in our model synchronous firing is dependent on the input stimulus but it is not necessarily locked to the input stimulus, that is, we have a stimulus-related synchronization [28]. Sideways gap junction connections induce synchronous firing. This is in line with evidence reported by Singer and Gray [29]. Our model also only uses local connections between neurons to establish synchronous firing. No global connections are required. Only a few models have been derived establishing synchronous firings using only local connections, for example, [30, 31]. Some models, however, require a global inhibitor to achieve desynchronization between different objects, for example, [32]. Schillen and König [33] use long-range excitatory delay connections in a network of nonlinear oscillators to achieve desynchronization. In our model, different firing rates, that is, desynchronization, are achieved through the size of the connected subnetworks. No global inhibitor is required. Subnetworks of different sizes will have different firing rates.

**Figure 3:**
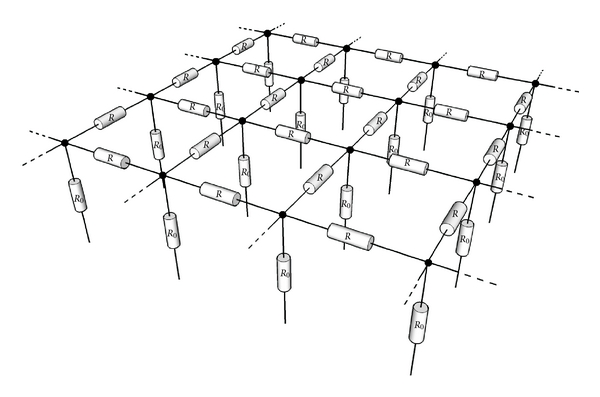
Resistive grid. Each node point is connected to another node point via a resistor *R*. An input current flows into the resistive grid through resistor *R*_0_.

In order to understand the function computed by a grid of resistively coupled neurons, let us consider the function computed by a resistive grid. In a resistive grid, neighboring points in a network are connected by resistors. We assume that an external current reaches each point of the network. Such a resistive grid is shown in Figure 3. Each node of the grid is connected via a resistor *R*. An input current is flowing into this resistive grid from below through resistor *R*_0_.

**Figure 4:**
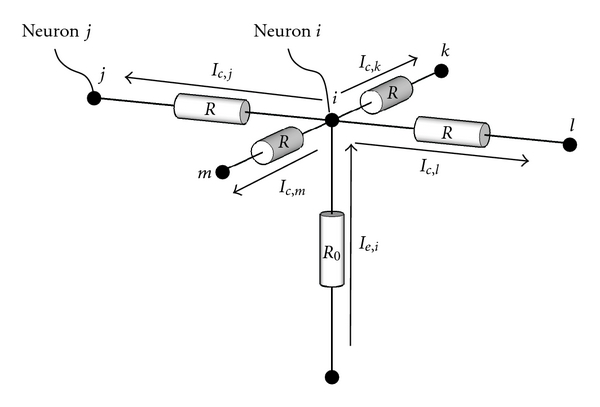
The external current *I*_*e*,*i*_ flowing into node *i* has to be equivalent to the current exchanged with adjacent nodes.

Each neuron corresponds to a point in this grid (see Figure 4). The external current *I*_*e*,*i*_ flowing into node *i*, that is, neuron *i*, is assumed to be a temporal integration of the output voltage of that same neuron. The external current has to be equivalent to the current exchanged with nearby neurons. Let *I*_*c*,*j*_ be the current exchanged with neuron *j*. Let neuron *i* be connected to *N*_*n*_ other neurons, then we have

(6) $$I_{e,i}=\overset{N_{n}}{\underset{j}{\sum }}I_{c,j}.$$

Let *V*_*e*,*i*_ be the input voltage and let *V*_*n*,*i*_, be the voltage at node *i*, then we obtain

(7) $$\frac{1}{R_{0}}\left(V_{c,i}-V_{e,i}\right)=\frac{1}{R}\overset{N_{n}}{\underset{j}{\sum }}V_{c,j}-\frac{N_{n}}{R}V_{c,i}$$

or

(8) $$V_{c,i}=\frac{R_{0}}{N_{n}R_{0}+R}\overset{N_{n}}{\underset{j}{\sum }}V_{c,j}+\frac{R}{N_{n}R_{0}+R}V_{e,i}.$$

We can rewrite this equation as

(9) $$V_{c,i}=\left(1-\alpha_{s}\right)\frac{1}{N_{n}}\overset{N_{n}}{\underset{j}{\sum }}V_{c,j}+\alpha_{s}V_{e,i}$$

with *α*_*s*_ = *R*/(*N*_*n*_ *R*_0_ + *R*). This operation again describes an averaging operation. First the spatial average of neighboring neurons is computed, and then this average is again averaged, adding a little from the external potential.

If we assume that we only have a linear sequence of neurons where each neuron is connected to its nearest neighbor then the solution of this equation is [34, 35]

(10) $$V_{c}\left(x\right)=\int \frac{1}{2\sigma }e^{-\left|x\right|/\sigma }V_{e}\left(x\right)dx$$

with $\sigma =\sqrt{\left(1-\alpha_{s}\right)/4\alpha_{s}}$. Note that we have dropped the index *i* and refer to both the input voltage *V*_*e*_ and the voltage *V*_*c*_ of neuron *i* through the position *x* of the neuron in the lattice. For a two-dimensional grid of neurons, parameterized by coordinates *x* and *y*, we can approximate the function computed by each neuron as

(11) $$V_{c}\left(x,y\right)=\int \int \frac{1}{4\sigma^{2}}e^{-\left(\left|x|+|y\right|\right)/\sigma }V_{e}\left(x,y\right)dx\;dy.$$

**Figure 5:**
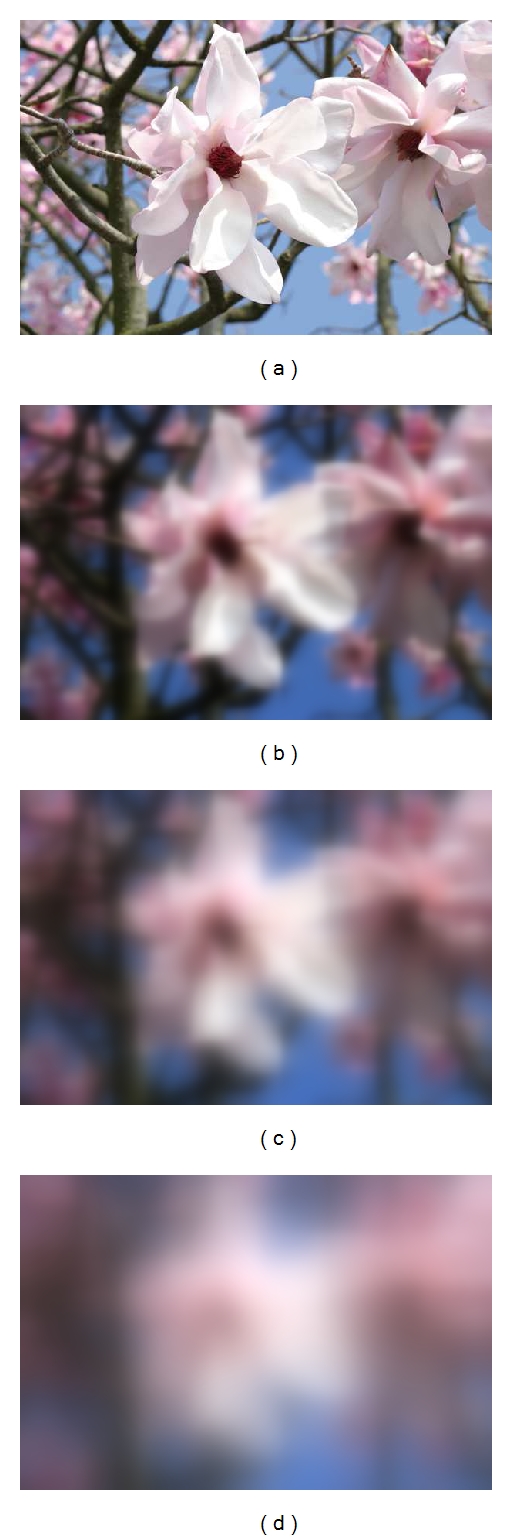
(a) Input image, (size 614 × 410) (b–d) spatially averaged images (b) *α*_*s*_ = 0.005, (c) *α*_*s*_ = 0.001, (d) *α*_*s*_ = 0.0002.

Figure 5 shows the result of this operation for different values of *α*_*s*_, respectively, *σ*. The input image is shown in Figure 5(a). Output images for *α*_*s*_ = 0.005, *α*_*s*_ = 0.001, and *α*_*s*_ = 0.0002 are shown in Figures 5(b)–5(d) where we have assumed that the grid of neurons processing the image has exactly the same size as the input image, that is, one neuron per pixel. Each neuron is assumed to be connected to its nearest neighbor. If *α*_*s*_ is very small, that is, the resistor *R* is very small compared to the input resistance *R*_0_ then a spatial average with a very large extend is computed. For *α*_*s*_ → 0 we obtain

(12) $$V_{c,i}=\frac{1}{N_{s}}\underset{j}{\sum }V_{c,j},$$

where *N*_*s*_ is the number of neurons in the resistively coupled network, that is, the network essentially computes the average of the node voltages for a sufficiently small value of *α*_*s*_.

## 4. A Functional View of Neural Computation through Sideways-Connected Spiking Neurons

A neuron is said to fire when the activation rises above a certain threshold. The integrate-and-fire model includes as parameters the strength with which the axon of a neuron is connected to the dendrites of the following neuron and the threshold. A common learning theory for the adjustments of the weights is Hebbian learning [36]. According to this theory, the connections between two neurons increase if both neurons are activated strongly. This allows tuning the neurons to many types of different stimuli, that is, the neuron fires strongly if the learned input is present. In computational data processing, use of a threshold is often a difficult issue. It is difficult to set the threshold at the right level to extract the relevant data. An adaptive threshold is often more appropriate and also more robust.

In the context of neural information processing, it is not clear how a suitable threshold is set. If the threshold is too high then hardly any neurons will fire. If the threshold is too low, then almost all neurons will fire all of the time. The threshold has to be within a suitable range for the neuron to function. The firing threshold for cortical neurons appears to vary spike to spike [37]. We assume that the threshold, which is used to extract relevant information, is determined by feeding back the output of a neuron. This allows adaptive tuning of the neuron to relevant information.

Our model actually uses two thresholds [38]. The first threshold is simply the standard threshold voltage. After the activation has reached this threshold voltage, the neuron fires. We will call this threshold the firing threshold. It can be set to an arbitrary but constant value. The second threshold which we introduce is the threshold which controls whether the gap junctions are open or closed. Traub et al. [39] work with a voltage-dependent behavior of the gap junctions. They used physical intuition rather than biological data to model this dependency. However, they do state that there appears to be a sharp threshold conductance, below which there is no synchronizing role of the interneuron dendritic gap junctions. In our model, the behavior of the gap junctions is also voltage dependent. Since the gap junctions control whether the neurons synchronize or not, we will call this second threshold the sync-threshold.

We assume that the sync-threshold is determined adaptively based on the firing rates of other neurons with a related function. Neurons with a related function are connected through gap junctions. The resistively coupling to other neurons enables the neuron to compute a spatial average of the output of other neurons. The neuron will “know” how active the other neurons are, and it is therefore able to tune its activity with respect to the firing rate of related neurons. We argue that the spatial and temporal average of the outgoing spikes of neurons with related functions is used to set the sync-threshold controlling the gap junctions. This allows the system to perform figure/ground segmentation.

For figure/ground segmentation, one needs to signal that several neurons actually respond to the same object, that is, that they respond to the same stimulus. According to our theory, this is achieved through gap junctions. We propose that gap junctions open when the temporal average of a neuron is above the spatial average of its output. In addition, we assume that the firing threshold of a neuron is influenced by the number of other neurons it is connected to through gap junctions. In our model, we actually vary the firing threshold based on the size of the connected network created through open gap junctions. Instead of varying the threshold, it is of course clear that varying the activation achieves the same result. 

We have used a varying threshold in our computational model that we describe in detail below. For the actual neuron it seems more likely that the firing threshold stays constant but the activation is increased (possibly by ions entering the neuron at positions where open gap junctions are located). Let *N*_*s*_ be the number of neurons responding to a certain stimulus. Then the firing threshold of each neuron responding to this stimulus is assumed to be reduced by *γN*_*s*_. As a result, neurons which respond to large objects will fire with a higher frequency, and, hence, the output will be treated as more relevant in further processing. This is in line with analyses of the behavior of biological neurons that stimulus-related information is encoded into the precise timing of spikes [40]. 

**Figure 6:**
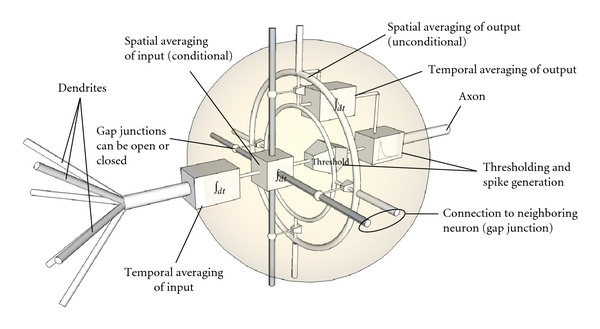
Artificial neuron with lateral connections (gap junctions). The operation of this model is fully specified by the algorithm given in Algorithm 1.

Our neuron model which also includes the function of gap junctions is illustrated in Figure 6. The full description of this model is given in Algorithm 1. But first, let us briefly describe the individual components of the model so that we get an overview. The comments in brackets refer to the illustration shown in Figure 6.

- Each neuron computes the temporal average of the incoming spikes through the afferent (∫*dt*-box).
- It fires if the temporal integral of the incoming spikes is larger than the firing threshold (threshold-box).
- Each neuron is part of two resistive grids (formed through light and dark lateral connections).
- A fixed resistive grid is formed by neurons connected through gap junctions (light lateral connections).
- A reconfigurable resistive grid or sub-network is formed by neurons connected through open gap junctions (dark lateral connections).
- Outgoing spikes are temporally integrated and spatially averaged using the fixed resistive grid (upper ∫*dt*-box and light lateral connections).
- This spatial average, essentially a feedback signal from the neuron's output, determines the sync-threshold of the neuron.
- Gap junctions to neighboring neurons open if the temporal average is larger than the spatial average otherwise they close, forming a reconfigurable resistive grid. A resistor exists in this grid for every open gap junction (sphere on dark lateral connection).
- Open gap junctions allow the neurons of a sub-network to synchronize (synchronization occurs through spatial integration ∫*dx*-box).
- The firing threshold of each neuron is reduced based on the size of the sub-network to which the neuron belongs.

Making the sync-threshold dependent on the spatial average of the output causes the threshold to move with the signal and allows for figure/ground separation.

Even though, in our model, all of the above functions are integrated into one neuron, it could actually be that some of the functions are spread over several different types of neurons within a cortical column. For a review of the columnar organization of the neocortex, see Mountcastle [41].

With this basic description of the function of a neuron, we are able to build a highly successful figure/ground separator or rather object detector. We will show this on some sample visual input.

## 5. A Detailed Example

Suppose that our model is used in the context of visual figure/ground segmentation. We start with an initial layer of neurons (the visual receptors). For our implementation, we only consider cones. The cones respond to light predominantly in the red, green, and blue parts of the spectrum [42]. Thus, for color image processing, we start off with a three-dimensional coordinate system. The coordinate axes are the responses of the cones in the red, green, and blue parts of the spectrum.

By the time the visual stimulus has reached the visual cortex, that is, V1, a change of coordinate system has occurred. The main axes are no longer red, green, and blue but dark-bright, red-green, and yellow-blue [43]. This transformation is due to so-called color opponent and double-opponent cells. Mathematically, the transformation is simply a rotation of the coordinate system [44]. For our simple example, we are only going to use the dark-bright channel. In order to simulate this channel, we compute the lightness [45] of the input stimulus for every pixel of the virtual retina. Let *R*, *G*, *B* be the nonlinear intensities stored in a computer image representing the responses of the red, green, and blue cones, then the lightness *L* is given as

(13) L=0.299·R+0.587·G+0.114·B.

We simulate a three-dimensional sheet of 1000 neurons which simulate the processing done by some, as of now, unspecific area of the visual cortex. The processing we describe could take place in V1. However, it seems that humans are not aware of the processing occurring in V1 [46]. The processing is more likely to take place in some higher visual area in particular if higher features such as form or motion are used.

**Figure 7:**
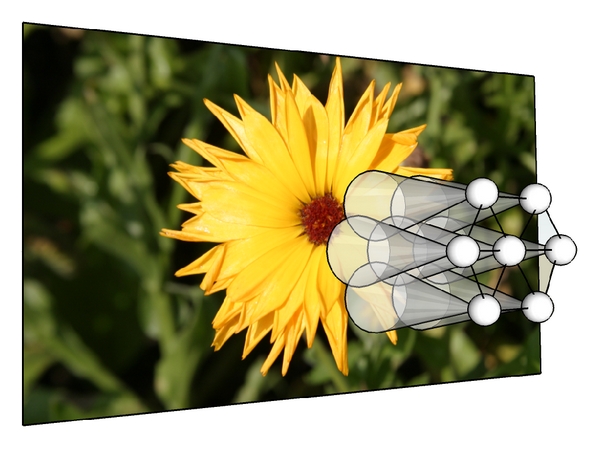
A set of neurons receives visual input from a virtual retina. Each neuron (sphere) has its own receptive field (transparent cone) and is resistively coupled to other neighboring neurons (connections between spheres). Only 7 neurons of a much larger set are shown. Axons are not shown.

In our simulation, each neuron has a random position inside a volume of size *N* = 100 × 100 × 10 units. Each neuron receives its input from three neurons of the virtual retina. The size of the retina is 614 × 410 pixels. The nonuniform distribution of the retinal receptors is not modeled. In the brain, the nonuniform distribution creates a complex-logarithmic mapping from the retinal receptors to the neurons of V1 [47, 48]. However, we are only concerned with the behavior of laterally connected neurons. The distribution is not relevant in this context. Thus, we simulate the receptive field as shown in Figure 7. Each neuron is laterally connected to its 6 nearest neighbors. The position where the neuron receives its input from is determined randomly by first mapping the position of the neuron to the virtual retina and then varying the position slightly (by one pixel to the left or right or up or down). The input we use is equal to the lightness of the pixel at that point of the retina. We do not simulate the spiking behavior of the retina as the first processing stage of the simulated sheet of neuron performs a temporal averaging anyway. The input may as well be simulated as a spiking input.

Each neuron is described by a set of state variables (shown in Table 1). The output *o* of a neuron is assumed to have the operating range of [0,1] and the activation *a* of a neuron is assumed to have the operating range of [−1, 1]. The algorithm describing how these state variables change over time is shown in Algorithm 1. In our simulation on a sequential computer, all of the neurons are updated sequentially. Note that the neurons are randomly distributed. Hence, the update is analogous to a random update on a grid array of neurons. The entire system takes approximately 1250 iterations before convergence to normal operating range using the parameters given in Figure 2.

**Figure 8:**
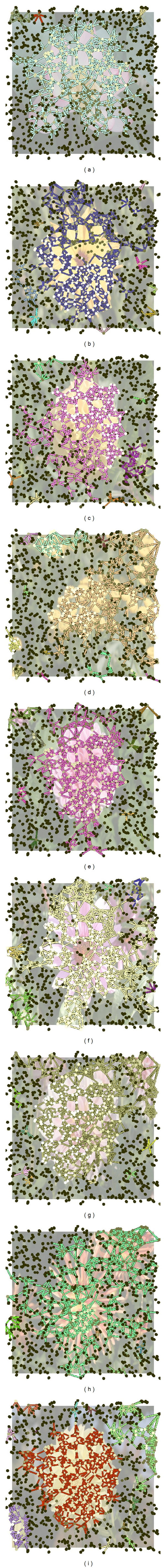
Figure/ground separation using our model. A sheet of neurons (nodes) receives input from a virtual retina (image in background). Gap junctions (connections between nodes) open if the temporal output of a neuron is above the spatial average.

Figure 8 shows how our sheet of neurons responds to different visual input stimuli. The input stimuli are shown in the background. Each node represents a neuron. The gap junctions open if the temporal average output is above the spatial average of the output of all neurons. Gap junctions are shown as connections between nodes. Only open gap junctions are shown in Figures 8(a)–8(i). The color of the neuron is drawn proportional to the temporal average of the neuron's output. The gap junctions of each interconnected sub-network is drawn with a different color. The color is randomly assigned but stays with a connected sub-network. The figure which has been separated from the ground can be clearly distinguished. 

Since open gap junctions connect adjacent neurons resistively, these subnetworks synchronize their firing rates in the same way that electrical circuits synchronize which are coupled resistively. It is almost certain that biological neurons are not all identical. They could even fire in a chaotic way. From the literature on electrical circuits, it is well known that chaotic circuits can be synchronized if a signal is sent from one circuit to the next [49, 50]. Also, identical non-linear electrical circuits have been shown to synchronize via bidirectional and unidirectional resistors [51]. Zhao and Breve [52] have shown that chaotic oscillators, in particular Wilson-Cowan neural oscillators [53], can be used for scene segmentation. In Zhao and Breve's setup, neurons responding to the same object synchronize whereas neurons representing other objects are in another chaotic orbit, that is, their response is not regular. In contrast to their work, we do not work with chaotic oscillators. Zhao and Breve only used static input. They did not experiment with moving stimuli where neurons have to continuously synchronize to the same object. Eckhorn et al. [54] also established synchrony in a moving input but worked with two one-dimensional layers of neurons. Their approach uses long-range lateral connections between neurons.

**Figure 9:**
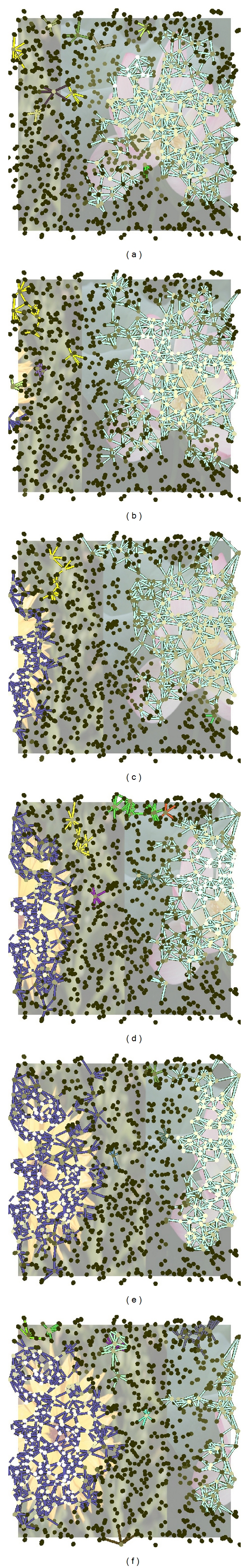
Figure/ground separation over several images from a larger image sequence. Every 100th image is shown. Even though a different set of neurons responds to the visual input, it is still the same connected sub-network as indicated by the color of the sub-network.

**Figure 10:**
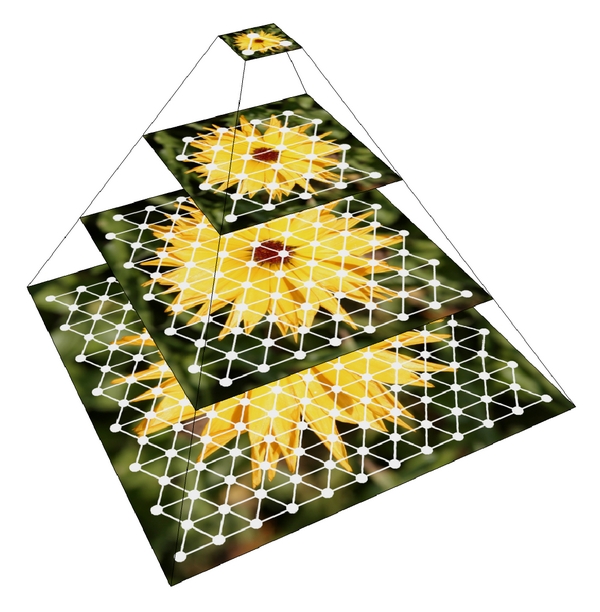
Hierarchy of neuron layers processing visual information at different scale levels.

Figure 9 shows that our method is able to follow the object over successive images of a moving stimulus. Even though a different set of neurons responds to this stimulus, it is still the same sub-network which is indicated by the color of the sub-network. The firing frequency will allow to identify this extracted stimulus as being the same object. With this information, the next stage of neurons is then able to compute the center of mass of this particular information, for example, using a hierarchy of neural layers as shown in Figure 10. This information in turn can then be used for tasks such as visual servoing [55, 56].

Rodemann [57] has shown that such gamma oscillations can be used as a temporal reference signal and also as a global processing switch. When gamma oscillations are used as a reference signal, neural processing can be changed from a rate encoding to a latency encoding allowing for faster information processing. With latency encoding, only the first spike and its exact timing within the cycle are relevant.

**Figure 11:**
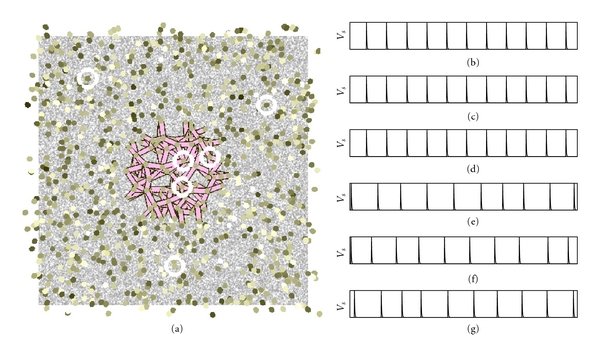
(a) Random input stimulus and small circular set of neurons with open gap junctions. (b)–(d) firing behavior of three neurons from inside the circular area. (e)–(f) Firing behavior of three neurons outside of the circular area.

We now further investigate the synchronizing behavior of our neural sheet of neurons using synthetic input.  Figure 11 shows how a set of neurons synchronize for an arbitrary random input and *γ* = 0.001. For this experiment, we deliberately open the gap junctions of all neurons which lie inside a circular area from the center of the sheet of neurons. In other words, all neurons inside the center area are resistively coupled to neighboring neurons whereas the remaining neurons are not resistively coupled to neighboring neurons. The input stimulus is defined as follows. At each time step, each input pixel is completely chosen at random from the range [0,1]. Thus, the input stimulus is just a sequence of random images without any kind of structure. The layer of neurons overlayed on a single input image is shown in Figure 11(a).

From the layer of neurons, three arbitrary neurons are selected from the center area and three arbitrary neurons are selected from the remaining neurons. The selected neurons are highlighted in Figure 11(a). Figures 11(b)–11(d) show the output of the three neurons from the center area. Figures 11(e)–11(g) show the output of the three neurons from the outside area. All neurons which are located inside the center area of the visual layer fire in synchrony. These neurons synchronize because they have their gap junctions open. The neurons which are located in the area outside of the circular area fire out of sync. The incoming random stimulus is summed up until the firing threshold is reached. For some neurons, the threshold will be reached soon whereas for others the firing threshold will be reached later. In the center area, the neurons are resistively coupled. Thus, the activation of all resistively coupled neurons will equalize to the same level (due to Algorithm 1(6)–( 8)).

Note that our model is in line with experimental results obtained by Lamme and Spekreijse [58]. They investigated whether neurons in V1 fire in synchrony depending on the position of their receptive field relative to the stimulus. They found that neurons tend to fire in synchrony if both of their receptive fields are located on either the object or on the background but not if one of the neuron's receptive field is located above the object and the other one over the background. Lamme and Spekreijse attribute this behavior to horizontal connections within V1. Apparently, whether or not the neurons of V1 synchronize depends on the type of stimulus used (and probably also on which neurons of V1 are checked for synchronous firing). There appears to be no synchronous firing behavior for a motion induced stimulus. This points to the use of higher visual areas, for example, V5 for figure/ground segmentation with respect to motion.

**Figure 12:**
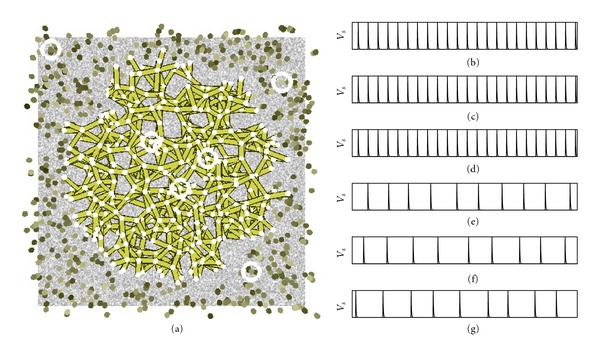
(a) Random input stimulus and large circular set of neurons with open gap junctions. (b)–(d) Firing behavior of three neurons from inside the circular area. (e)–(f) firing behavior of three neurons outside of the circular area.

We will now illustrate the effect of line (11) of the update algorithm (Algorithm 1) on the processing performed by the neurons. The firing threshold is reduced depending on the size of the sub-network, because of *γ* = 0.001. If many neurons are resistively coupled through open gap junctions, then their firing threshold will be lowered leading to a higher firing frequency. If just a few neurons are resistively coupled, then they will fire with a slower frequency. This effect is illustrated when comparing between Figures 11 and 12. For the small circular area shown in Figure 11, the firing frequency of neurons (b)–(d) is lower compared to the firing frequency of neurons (b)–(d) for the larger area shown in Figure 12.

Table 2 shows the parameters which we have used for these experiments. The parameters *α*_*o*_ and *α*_*a*_ describing the decay of the output spiking voltage *V*_*s*_ and the decay of the neuron's activation potential *V*_*i*_ of course depend on the time scale of the simulation. Similarly, the factor *α*_*t*_ for the temporal averaging of the neuron's output also depends on the time scale of the simulation. The parameter *α*_*s*_ which determines the extent of the spatial averaging should be reasonably small. This parameter depends on the total number of neurons *N* simulated in the sheet of neurons. The more neurons there are, the smaller this parameter has to be in order to compute an almost global average of the temporal average. The parameter *ϵ* determines how much from the built-up voltage carries over to adjacent neurons. This parameter is most likely very small as most of the current leaves the neuron through the axon. However, part of this current also reaches neighboring neurons. If those neurons have almost reached their threshold then this current will make sure that these neurons also fire at approximately the same time.

The factor *γ* which is used to reduce the firing threshold of a neuron is of course based on the maximum number of neurons which can belong to a connected set of neurons. Let there be *N* neurons in the simulated sheet, then this parameter should be smaller than 2/*N*. The maximum size of a sub-network is *N*/2. For such a maximum size sub-network, the firing threshold would be reduced to 0 if *γ* = 2/*N*, that is, all neurons of the sub-network would fire all of the time. On the other hand, if *γ* is too small, then the reduction of the firing threshold would hardly make a difference, and, hence, it would not be possible to distinguish between smaller or larger stimuli by higher neural areas. Our virtual retina, that is, the input images that we used, had size 614 × 410 pixels. The weights *w*_*ij*_ are set to unity. Each neuron receives its stimulus from the artificial retina as described before with a slight random offset. Due to the unit weights and this offset, the input is a downsampled version of the original retina. The resulting synchronous firing frequency of course depends on the choice of the given parameters and the simulation time constant. By varying the time constant of the simulation step, the firing frequency can be brought into agreement with a given firing frequency. Also, note that the so-called gamma synchrony does not correspond to a single frequency but to a range of frequencies. Our model also shows this behavior in that several different frequencies can be obtained as output.

## 6. Processing of Arbitrary Features in the Cortex

For our simulations we have used a retinotopic mapping between the neurons processing the visual input and the virtual retina. It is well known that the primary visual cortex is highly structured [59]. It is of course clear that the operation which we just described also works with nonretinotopic maps. The only requirement for the method described to work is that we interconnect neurons of related function resistively such that the spatial average can be computed and in turn the sync-threshold can be set.

**Figure 13:**
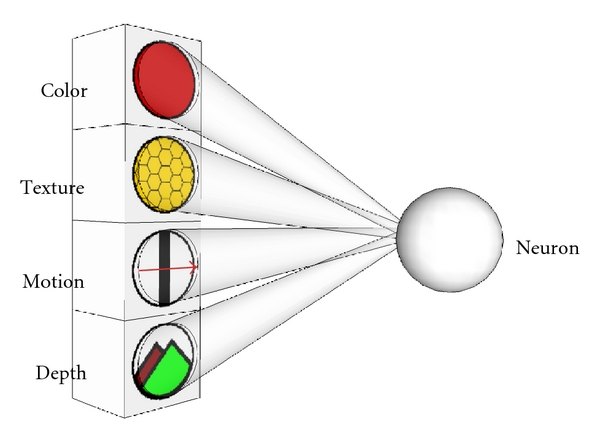
Our model is also able to work with more complex feature vectors. Instead of using only the lightness of the visual stimuli, one could also use color, texture, motion, or disparity. Also, the input does not have to be visual input. It could be any kind of input, for example, auditory input.

Even though we have shown how our model processes a very simple visual input (the lightness of the stimulus), the method is able to classify any arbitrary feature vector. If visual stimuli are processed, the component features could as well be color, texture, motion, or depth (derived from disparity) as shown in Figure 13.

Suppose that one wants to segment a moving stimuli from a background motion. Let us assume that the moving object creates a different motion vector compared to the background. Then it would be sufficient to extract this object by substituting the lightness input (13) with a motion detector.

Suppose that two different stimuli are presented to our layer of neurons, for example, two objects which move through space. Then the lightness input would be substituted by a motion detector and a texture detector tuned to the object. Neurons corresponding to the object covering a larger retinal area would fire with a higher firing frequency. Neurons which correspond to the smaller object would have a lower firing frequency. Using a hierarchy of frequency detecting neurons [60], we could locate the position of the object relative to the visual field. This information could then be used for actions such as grasping behavior through visual servo control [55, 56].

## 7. Discussion

Kouider [61] reviews current neurobiological theories for consciousness. Unfortunately, these theories are not constructive in a way that would allow engineers to build a conscious machine or artifact. We review a few of these theories and show how they relate to our model.

Tononi and Edelman [62] put forward the dynamic core hypothesis in which information is transmitted through recurrent reentry connections along an ascending thalamo-cortical axis of arousal. Local groups of neurons perform specialized and discriminatory functions regulating reentry and feedback. Particular core feedback loops are presumed to correspond with particular conscious mental states. Cortical feedback between different visual areas is important for figure/ground separation [63], and, according to Grossberg [64], visual form perception.

But the dynamic core cannot account for gamma synchrony EEG, the best marker of consciousness, nor deal with non-arousal-based consciousness, that is, internally generated states like daydreaming, mind-wandering, memory, and meditation, mediated through “default-mode” networks [5]. Thalamic core activity could be essentially nonconscious, unless enveloped within a synchronized zone, conferring (by an as-yet-unknown mechanism) conscious awareness of its content [15].

In the present paper we consider just local zones of gap junction-mediated synchrony, able to move through neuronal network lateral connections. Such local zones could, for example, regulate reentry and feedback in the dynamic core. We did not consider long-range gap junction connections which may occur via interneurons, glia and axonal gap junctions, coalescing mobile zones into synchronized global webs.

According to Tononi's Information Integration Theory [65] consciousness depends exclusively on the ability to integrate information, to reduce uncertainty. The quality of consciousness is determined by complexity of relationships among informational elements. His theory also suggests an ability to measure and correlate consciousness with the brain's electrical complexity. In integrate-and-fire neurons, integration occurs exclusively in dendrites and cell body, axon firing being the output signal. But Tononi integration occurs in intracortical pathways over large regions of cortex and thus linear series of individual integrate-and-fire neurons. In our model, integration-performing dendrites and cell bodies are synchronized and unified by gap junctions into lateral webs, enabling, we propose, faster and more efficient “collective integration” by massive parallel processing of synaptic inputs from among many thousands of neurons, with more finely tuned and coordinated firing outputs.

Dehaene and Naccache [66] have developed the global neuronal workspace theory. It assumes that different modular areas, including prefrontal and anterior cortex, are connected through long-range axons into a “global workspace,” within which consciousness can occur in a further subset of neuronal activities. Our mobile zone of synchrony defined by lateral gap junction could easily move through the global workspace, conferring consciousness wherever it goes.

Lamme [67] has put forward the Local Recurrence Theory, a hierarchy of three types of neural processes, (1) a feed-forward sweep, (2) localized recurrent processing, and (3) widespread recurrent processing with global interactions. All seem conducive to gap-junction-mediated mobile zones and more extended global webs.

The Microconsciousness Theory of Zeki [68] suggests that particular qualities of a perception become conscious in separated brain areas, with multiple microconsciousnesses distributed across processing sites. Attributes such as color, form, and motion each arise in one particular microconsciousness region, but are somehow bound together to give rise to a unified conscious percept. Our model of a mobile zone of synchrony is a direct correlate of microconsciousness. Zeki does not explain how the microconsciousnesses are bound together. They may need long-range gap junctions (interneurons, glia, axons) and brain-wide mobile zones/global webs for binding.

Binding is an essential question. How does the brain integrate sensory inputs, binding together individual features from different cortical areas into unified, conscious percepts? If individual neurons were tuned to specific stimuli, many highly specialized cells would be required which would only fire rarely, since relevant stimuli only appear on occasion. As a solution to this problem, von der Malsburg [69] proposed the “correlation theory,” in which synchronous electrical activity among disparate cell groups binds them together and integrates their component features into a unified conscious perception. According to this theory, relations between active cells leading to synchrony are established by synaptic modulation and feedback loops. Wang et al. [70] showed how a feedback loop between groups of excitatory and inhibitory neurons can be used for pattern segmentation in associative memory. Gerstner et al. [17] and Ritz et al. [71] showed how such feedback loops can establish collective oscillations. Using this architecture, von der Malsburg and Buhmann [72] presented a computational model of a cortical circuit consisting of an array of synchronized units that act as feature detectors.

Synchrony in the gamma EEG range of 30 to 90 Hz, correlating with conscious perceptions and actions, was discovered and established in the 1980s for example, Singer [73] gives an extensive overview on brain gamma synchrony correlating with perception and motor control. Palva et al. [74] showed robust cross-frequency (alpha, beta and gamma oscillations) phase synchrony exists in human cortex, with synchrony enhanced during cognitive tasks such as arithmetic.

However several influential papers discounted synchrony as a solution to binding or consciousness, based on a misunderstanding. Von der Malsburg had implied axonal firings, or spikes, as the synchronized activity, and neuroscientists and cognitive scientists routinely view firings or spikes as the currency of cognition in the brain. However gamma synchrony EEG correlating with consciousness measures local field potentials, closely related to dendritic and cell body membrane potentials rather than axonal firings.

In a famous 1990 paper, Crick and Koch [75] argued that consciousness depends on neurons that bind together by synchronizing their spikes in 40 Hz oscillations. However 5 years later, as evidence for synchronized spikes failed to materialize (and despite continuing evidence for gamma synchrony EEG, that is, dendritic synchrony as a neural correlate of consciousness) Crick and Koch [46] recanted their support for synchrony as an essential aspect of brain activity related to consciousness. Shadlen and Movshon [76] concluded there is insufficient evidence for the temporal binding hypothesis based on synchronized axonal firings. Forced to choose between dendritic synchrony (for which evidence existed) and axonal firings as the correlate of consciousness, authorities chose axonal firings, presumably because of their direct applicability to neuronal network computation.

But integration, which Tononi tells us is the key function relating to consciousness, occurs in post-synaptic dendrites and cell bodies. Gamma synchrony EEG originates in postsynaptic dendrites and cell bodies. Gap junction-connected mobile zones of dendritic synchrony performing collective integration are prime candidates for the neural correlate of consciousness.

Crick and Koch [75] and Shadlen and Movshon [76] both also questioned whether synchronized oscillations could solve the figure/ground problem. In this paper we present an algorithmic solution to the figure/ground problem based on dendritic synchrony. Specifically, we demonstrate a spatiotemporal envelope of sideways synchrony moving through a single-layer artificial neural network viewing and perceiving a visual scene. Topology of the envelope and activity within it convey information, not the synchrony *per se*. Neurons of related function, connected through gap junctions, synchronize and coherently respond to an input stimulus. This is in line with evidence summarized by Singer and Gray [29], that is, that correlations tend to occur between cells with similarities in orientation preferences, ocular dominances, and color selectivities.

Singer and Gray, as well as Crick and Koch, Shadlen and Movshon, Tononi, Edelman, and Lamme based their models on axonal-dendritic synapses, with synchrony and long-range correlations due to axonal firing/synaptic feedback loops along sensory arousal pathways. Generally, they all accommodate nonconscious cognitive processes and behaviors, but fail to offer a distinction for consciousness.

The importance of gap junctions in the brain, and in particular in relation to gamma synchrony, was not then appreciated. Our model of a gap-junction-mediated mobile sub-network, zone, or envelope of dendritic synchrony moving through input/integration layers of neuronal synaptic networks is compatible with, and supplementary to all these models, capable of adding to them a distinguishing mechanism for consciousness.

## 8. Conclusion

Cognitive brain functions are understood as computation in synaptic networks of integrate-and-fire neurons. Each neuron has multiple dendrites and a cell body which integrate synaptic inputs to a threshold triggering axonal firings or spikes. With feedback and synaptic modifications, networks of such neurons learn, adapt, and compute, able to account for cognitive functions. Axonal firings or spikes and chemical synaptic transmissions are considered the primary currency of cognitive information processing in the brain's neuronal networks. But a basis for consciousness in the brain remains elusive, as does executive agency in artificial systems based on neuronal networks.

At the same time, another type of synaptic network occurs among brain neurons. Gap junctions (electrical synapses) fuse adjacent cells, synchronize their membranes and connect their cytoplasms, essentially forming subnetworks which are one complex cell, syncytium, “hyper-neuron,” or dendritic web. Gap junction-connected subnetworks among cortical interneurons mediate gamma synchrony EEG, the best measurable correlate of consciousness. Gap-junctions between dendrites form lateral, or sideways envelopes, or layers in neuronal networks. As gap junctions open and close, zones or webs of gap junction-connected neurons and glia can literally move around the brain, as an envelope of synchronized collective integration, perhaps able to confer conscious awareness upon its contents [15]. If consciousness moves as a self-organizing system through the brain's neuronal networks, perhaps a comparable function could be engineered into artificial systems. In this paper we applied the concept to an artificial neural network.

We extend the standard integrate-and-fire neuronal model in an artificial system to include “sideways synchrony” induced by lateral connections in input/integration layers. In distinguishing “figure” from “ground” in visual signals, neurons extract essential features from an input stimulus. In our computational model, we introduce lateral processing through gap junctions which couple neurons of similar function. Each neuron temporally integrates its own inputs to a threshold which, when met, results in its own output spike. The generated output spikes are used as a feedback signal for the same neuron. This feedback signal is then averaged over gap-junction-connected neighboring neurons, regardless of whether the gap junctions are open or closed. Neurons with a firing frequency above the spatial average open their gap junctions with neighboring neurons, causing these coupled neurons to synchronize, providing coherent processing from one time step to the next. Opening and closing of gap junctions enable the sub-network of gap-junction-connected cells to literally move through the larger network.

Due to coherent processing and collective integration, the sub-network of synchronized neurons may be more efficient. In the brain, according to our view, gap-junction-defined synchronized zones correlate with conscious perception and control, converting nonconscious cognition to consciousness. In artificial systems, a synchronized zone can act as a mobile executive, a causal agent. This study demonstrates the potential utility of a mobile synchronized zone in feature detection and visual perception. Our mobile zone of synchrony is a candidate for (1) the neural correlate of consciousness in the brain and (2) an executive causal agent in artificial systems.

## Figures and Tables

**Algorithm 1:**
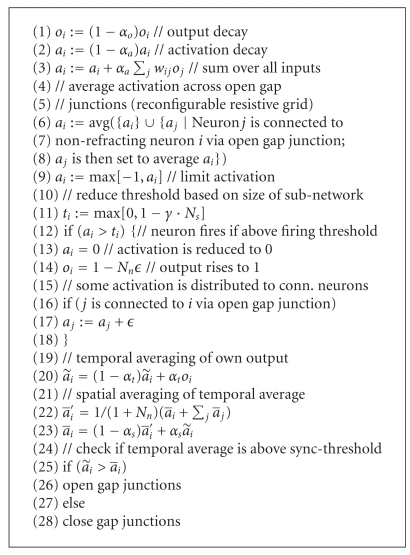
Algorithm which updates the state variables of neuron *i* from one time step to the next.

**Table 1:** State variables of neuron *i*.

Variable	Physical correlate	Description
*o*_*i*_	*V*_*s*_	Output voltage sent along axon
*a*_*i*_	*V*_*i*_	Activation of neuron
*t*_*i*_	*V*_threshold_	Firing threshold voltage
${\tilde{a}}_{i}$	*V*_*e*,*i*_	Temporal average of outgoing spikes
${\bar{a}}_{i}$	*V*_*c*,*i*_	Spatial average of temporal average

**Table 2:** Parameters used for the simulation.

Parameter	Description	Value
*α*_*o*_	Decay of the output spiking voltage	0.5
*α*_*a*_	Decay of the neuron's activation potential	0.01
*α*_*t*_	Temporal averaging factor	0.01
*α*_*s*_	Spatial averaging factor	0.0001
*ϵ*	Activation leakage to adjacent neuron upon firing	0.001
*γ*	Factor influencing reduction of firing threshold	0.0
*w*_*ij*_	Weight between neurons *i* and *j*	1
*o*_*j*_	Output of retinal neuron	*L*(*x*, *y*)
